# Histological analysis of thrombus composition in acute ischemic stroke with large vessel occlusion: impact of r-tPA treatment

**DOI:** 10.3389/fnins.2025.1707934

**Published:** 2025-11-21

**Authors:** Changzhi Zhao, Guofang Chen, Zhiliang Guo, Sha Chen, Lei Ping, Qiao Li, Xiaoying Zhang, Manhua Ding, Yang Zhang

**Affiliations:** 1 Department of Neurosurgery, Xuzhou Central Hospital, Southeast University Affiliated Xuzhou Central Hospital, Xuzhou Clinical School of Xuzhou Medical University, Xuzhou, China; 2 Department of Neurology, Xuzhou Central Hospital, Southeast University Affiliated Xuzhou Central Hospital, Xuzhou Clinical School of Xuzhou Medical University, Xuzhou, China; 3 Department of Neurology and Suzhou Clinical Research Center of Neurological Disease, The Second Affiliated Hospital of Soochow University, Suzhou, China; 4 Department of Radiotherapy, Xuzhou Cancer Hospital, Xuzhou, China

**Keywords:** ischemic stroke, thrombus, TOAST classification, mechanical thrombectomy, thrombolysis

## Abstract

**Background and purpose:**

Mechanical thrombectomy advances enable detection of thrombus composition changes post-intravenous thrombolysis, but evidence remains limited. This study retrospectively analyzed thrombi obtained from mechanical thrombectomy in patients with acute ischemic stroke to explore the impact of intravenous thrombolysis (IVT) on the composition of thrombi in patients with different TOAST classifications and the association between changes in thrombus components and clinical outcome data.

**Materials and methods:**

Thrombus samples from 141 patients undergoing mechanical thrombectomy (*n* = 60) or bridging therapy (*n* = 81) at two hospitals between December 2020 and January 2024 were included. Baseline data and outcome data were collected. Components were analyzed via HE staining and immunohistochemistry for CD61 and FGB, quantifying area percentages of red blood cell (RBC), white blood cell (WBC), platelet-fibrin complexes (PLT + FIB), platelet (PLT), and fibrin (FIB). Thrombus components were compared between the mechanical thrombectomy and bridging therapy groups according to TOAST subtypes, and a combined analysis was conducted with clinical prognosis and surgical conditions.

**Results:**

No significant baseline differences (*p* > 0.05). Significant component differences between mechanical thrombectomy and bridging therapy across subtypes: in large artery atherosclerotic (LAA), FIB higher in mechanical thrombectomy (*p* = 0.001); in cardioembolic (CE), PLT and FIB higher in mechanical thrombectomy (*p* = 0.003, *p* < 0.001). Subtype comparisons showed differences: LAA vs. CE in RBC, PLT + FIB, PLT, FIB (*p* < 0.05); LAA vs. stroke of undetermined source (SUE) in PLT and FIB (*p* < 0.05); CE vs. SUE in FIB (*p* < 0.05).

**Conclusion:**

This study quantified the thrombus components of different etiologies and found that IVT could reduce the FIB content in thrombi of large artery occlusion cerebral infarction, especially in the LAA type. The platelet content in the LAA type was higher than that in the CE and the SUE types. The FIB level in the SUE type was higher than that in the CE type. FIB was not only present in the PLT area but also in the RBC area. IVT may improve thrombectomy success by disrupting thrombus structure, but it can increase fragment embolization and prolong procedure time.

## Introduction

Prior to 2015, as the mechanical thrombectomy (MT) technology remained imperfect and its therapeutic effectiveness had not been extensively validated, there was a paucity of research focusing on the pathological characteristics of thrombi (Staessens et al., 2021). Along with the progress of technology and the publication of the results of pivotal clinical trials, MT has currently emerged as the mainstay treatment for acute large-vessel occlusive ischemic stroke (AIS-LVO). Furthermore, while MT exhibits a higher vascular recanalization rate compared to intravenous thrombolysis (IVT), IVT remains the preferred choice for patients who meet the time-window criteria, attributable to its operational simplicity and relatively low cost (National Institute of Neurological Disorders and Stroke rt-PA Stroke Study Group, 1995; Hacke et al., 2008). Nevertheless, a subset of patients with stroke resulting from large vessel occlusion (LVO) demonstrate a poor response to IVT and subsequently undergo MT treatment (Gong et al., 2019). This combined treatment modality is referred to as bridging therapy (BT). Several studies have compared the efficacy of MT and BT with the aim of investigating whether IVT exerts an influence. These studies reported that BT can enhance the vascular recanalization rate (Behme et al., 2016). However, the subgroup analyses of SWIFT and STAR, as well as the HERMES meta-analysis, did not lend support to this finding (Goyal et al., 2016; Coutinho et al., 2017). We considered that the inherent bias could potentially stem from the inclusion of MT patients who did not meet the eligibility criteria for IVT. Additionally, research has indicated that among patients who meet the IVT criteria, there are no significant differences in clinical outcomes between those who undergo MT and those who receive BT (Tsivgoulis et al., 2017). Therefore, the debate over whether IVT treatment is necessary before MT continues.

Although MT has increased the rate of vascular recanalization, there are still individual differences in clinical outcomes. Studies have shown that the histopathological composition of thrombi may be a key factor influencing the efficacy of thrombectomy (Yuki et al., 2012), and the composition of thrombi may also influence the thrombectomy strategy in endovascular treatment. In recent years, research on the association between thrombus components and the etiology of stroke has gradually deepened, especially in exploring the pathogenesis of intracranial AIS-LVO, significant progress has been made (Fitzgerald et al., 2021; Manisha et al., 2024). Staessens et al.’s (2020) research found that in AIS-LVO patients, the proportions of red blood cells, white blood cells, and fibrin in thrombi vary among different etiological subtypes. Moreover, thrombi with different components can affect the success rate of intravenous thrombolysis, the number of mechanical thrombectomy operations, the incidence of complications, and clinical prognosis. Niesten et al.’s (2014) research indicated that thrombi rich in fibrin are not only associated with a greater need for repeated recanalization operations during thrombectomy but also show greater resistance to thrombolytic therapy compared to those rich in red blood cells. Vandelanotte et al.’s (2024a) research found that thrombi rich in red blood cells are more sensitive to thrombolytic therapy. Zhang et al.’s (2024) research discovered that thrombi rich in red blood cells are associated with a shorter time interval from puncture to reperfusion in patients with acute ischemic stroke and better clinical outcomes.

Furthermore, the study by Rossi et al. (2021) found that the extracted thrombi from patients who received BT were significantly smaller than those from patients who only received MT, although the final results of vascular recanalization were not different. Currently, studies on the differences in thrombus composition between patients receiving MT and BT are relatively limited. Most previous studies only used one type of staining, such as hematoxylin–eosin staining (HE), Masson’s trichrome staining (MSB), or HE staining combined with immunohistochemistry (IHC). Few studies have simultaneously used multiple staining methods to investigate the components of thrombi (Aliena-Valero et al., 2021). There is a lack of research on the differences in thrombus composition among patients with different stroke subtypes who received MT and BT treatments. Data on the association between thrombus composition and stroke subtypes are still insufficient.

Therefore, this study aims to evaluate thrombus components in AIS-LVO patients using HE staining and IHC, assess the effect of IVT on thrombus composition, and correlate findings with clinical outcomes and surgical characteristics to provide insights into stroke pathogenesis.

## Materials and methods

### Enrollment of patients

This study was a retrospective one, consecutively enrolling AIS-LVO patients who were treated with MT at Xuzhou Central Hospital and the Second Affiliated Hospital of Soochow University from December 2020 to January 2024. Patients were divided into the BT group (*n* = 81) and the MT group (*n* = 60) based on whether they received IVT treatment or not.

### Inclusion and exclusion criteria

Inclusion Criteria: (1) Age≥18 years old; (2) Time of onset≤24 h; (3) All received MT and successfully removed the thrombus; (4) Preoperative Modified Rankin Scale (mRS) score≤2 points; (5) Preoperative National Institutes of Health Stroke Scale (NIHSS) score≥6 points; (6) Preoperative Alberta Stroke Program Early CT Score (ASPECTS) ≥ 6 points.

Exclusion Criteria: (1) Preoperative CT indicated cerebral hemorrhage; (2) Patient lacks clinical or imaging data; (3) Patients with contraindications related to thrombolysis; (4) Patients with severe dysfunction of heart, liver, spleen, lung or kidney, or malignant tumors, making them ineligible for thrombectomy; (5) Patients not falling into any of the LAA, CE or SUE categories of the TOAST classification.

### Collection of baseline data

Collect the baseline data of patients, including age, gender, vascular risk factors (previous history of coronary heart disease, atrial fibrillation), laboratory tests (coagulation indicators, blood lipids), etiological classification of stroke, preoperative NIHSS score, anterior and posterior circulation, preoperative mRS score, time from admission to femoral artery puncture (Door-to-puncture time, DPT), and time from onset to vascular recanalization (onset-to-recanalization time, ORT).

### Collection of data on clinical outcomes and surgical characteristics

Data were collected on puncture-to-recanalization time (PRT), 90-day favorable functional outcome (defined as mRS ≤ 2), 90-day mortality, successful revascularization (mTICI≥2b), number of thrombectomy passes, first-pass efficacy, and symptomatic intracranial hemorrhage (SICH). SICH was defined as any parenchymal hematoma, subarachnoid hemorrhage or intraventricular hemorrhage associated with death, or a neurological deterioration of ≥ 4 points on the NIHSS within 24 h.

### Ethics

All patients received informed consent signed by the patients or their legal representatives before intravenous thrombolysis or endovascular mechanical thrombectomy. This study was approved by the Ethics Committee of Xuzhou Central Hospital and the Second Affiliated Hospital of Soochow University (Ethics Number: XZXY-LK-20230517-074, JD-LK2023070-I01).

### Intravenous thrombolysis and mechanical thrombectomy

Patients who met the indications for intravenous thrombolysis and gave informed consent were treated with 0.9 mg/kg alteplase (r-tPA) intravenously (Emberson et al., 2014). The patients underwent MT, and the thrombus was collected in a sterile manner.

### Analysis of histological components

First, the thrombus was immediately placed in a universal tissue fixative (10% formalin solution) for more than 24 h after removal and then stored and transported at room temperature. The tissue was taken out of the fixative and trimmed flat with a scalpel in a fume hood at the target site. The trimmed tissue and corresponding labels were placed in embedding frames and put into a dehydrator for sequential dehydration with gradient alcohol.

Secondly, consecutive sections of thrombus with a thickness of 3 micrometers were used (i.e., obtained from the same thrombus sample) to ensure the comparability of staining results. These sections were stained with hematoxylin–eosin (HE) to observe the distribution and morphological characteristics of red blood cells (RBC), white blood cells (WBC), and platelet-fibrin complexes (PLT + FIB). Using CD61 IHC staining to observe platelets (PLT) alone, and use FGB IHC staining to observe fibrin (FIB) alone. For immunohistochemical staining, paraffin sections were deparaffinized and rehydrated through a gradient of alcohol. The following steps were then carried out in sequence: (1) Antigen retrieval specific to the tissue (preventing excessive evaporation of the buffer solution and avoiding dry sections); (2) Blocking with 3% H₂O₂ (25 min, protected from light); (3) Blocking with serum (30 min, at room temperature); (4) Incubation with primary antibody CD61 (integrin β3, 66,952-1-ig, Proteintech, 1:1000) overnight at 4 °C; (5) Incubation with HRP-labeled secondary antibody (Anti-FGB Antibody, M01204-1, BOSTER, 1:200) for 50 min at room temperature; (6) Color development with DAB chromogen from the immunohistochemical kit followed by washing with water to stop the reaction; (7) Counterstaining with hematoxylin (3 min) and bluing with hematoxylin bluing solution; (8) Dehydration through a gradient of alcohol and clearing with xylene; (9) Mounting with neutral gum; (10) Analysis under an optical microscope. Between all steps, sections were washed with PBS buffer (3 × 5 min each time), and the duration and concentration of each chemical treatment were carried out according to the standard protocol. It should be noted that the serum blocking solution should be selected based on the species of the primary antibody [if the primary antibody is from goat, use rabbit serum for blocking, otherwise use bovine serum albumin (BSA)].

Finally, after all sections were examined under an optical microscope, a full-field digital section was generated using a slide digital scanner (Pannoramic MIDI), and then the scanned images were converted to digital images (TIFF format) using CaseViewer (C.V2.4, 3DHISTECH, Hungary) for analysis of thrombus components. All the relevant images of HE-stained specimens were analyzed by a pathologist who was unaware of the patients’ clinical information, with the magnification adjusted to 100 × . The area percentages of all stained red blood cells, fibrin and platelet complexes, fibrin, platelets and white blood cells were batch-calculated by a researcher who was unaware of the patient’s clinical and image information using the Trainable Weka Segmentation plugin in Fiji software (2.14, https://imagej.net/software/fiji) in ijm format. When using the Trainable Weka Segmentation plugin to analyze stained images, four color categories are set for target recognition. By default, two categories with customizable chromaticity coordinates are provided, and the two newly added categories use the default values. In HE staining: green [51, 255, 0] represents the PLT and FIB complex, blue [0, 0, 204] represents WBC, purple (default) represents RBC, and yellow (default) represents the background; in CD61 staining (for PLT): green [51, 255, 0] represents other complexes, blue [0, 0, 204] represents WBC, purple (default) represents PLT, and yellow (default) represents the background; in FGB staining (for FIB): purple (default) represents FIB, and the rest are consistent with CD61 staining.

### Criteria for the classification of stroke subtypes

The stroke subtypes were determined by two researchers based on The publications Committee for the Trial of ORG 10172 in acute stroke treatment (TOAST) investigators (1998) method, and they were blinded to each other during the assessment process. After the initial classification was completed, a third researcher checked the results. For cases with discrepancies, the final classification was determined by a senior physician. Large artery atherosclerosis (LAA) was defined when significant stenosis (>50%) was present in the large arteries associated with acute ischemic stroke and no evidence of potential cardiogenic embolism was found. Cardiogenic embolism (CE) was defined as the presence of at least one clear evidence of cardiogenic embolism source. Stroke cases with more than two causes or with an unclear cause despite patient evaluation were classified as stroke of undetermined source (SUE).

### Statistical analysis

Statistical analysis was performed using SPSS 27.0. The Kolmogorov–Smirnov test was used for normality testing. For data that conformed to a normal distribution, the mean ± standard deviation ( $\bar{x}$ ± s,) was used to represent the measurement data, and the independent samples t-test was used for comparison between two groups. For data that did not conform to a normal distribution, the median and interquartile range were used to represent the measurement data. The Mann–Whitney U test was used for statistical analysis in the comparison between different TOAST subtypes and within the MT group and BT group for each TOAST subtype. Count data were expressed as frequency and percentage, and the *χ*^2^ test or Fisher’s exact test was used for comparison between two groups. A *p* value < 0.05 was considered statistically significant.

## Results

### Baseline data

The baseline data of 141 patients included in the study are shown in Table 1. No statistically significant differences were found among the groups (*p* > 0.05). Among the 141 included patients, there were 36 cases (25.5%) of LAA, 63 cases (44.7%) of cardioembolic stroke, and 42 cases (29.8%) of undetermined etiology. The proportions of TOAST subtypes in the MT group and the BT group are shown in Table 1, and there was no significant difference in the composition of TOAST subtypes between the two groups (*p* = 0.915).

**Table 1:** Baseline data.

Indicators	All data (*n* = 141)	MT (*n* = 81)	BT (*n* = 60)	t/U/X^2^	*p*
Age (y; *M*, *IQR*)	69.00 ± 12.00	68.00 ± 12.00	70.00 ± 12.00	−0.712^a^	0.478
Male [*n* (%)]	83 (58.87)	48 (59.26)	35 (58.33)	0.012^c^	0.912
Hypertension [*n* (%)]	91 (64.5)	54 (66.67)	37 (61.67)	0.377^c^	0.539
Diabetes [*n* (%)]	28 (19.9)	19 (23.46)	9 (15.00)	1.549^c^	0.213
Atrial fibrillation [*n* (%)]	66 (46.81)	36 (44.44)	30 (50.00)	0.427^c^	0.513
Coronary artery disease [*n* (%)]	45 (31.91)	26 (32.10)	19 (31.67)	0.003^c^	0.957
Previous dual-antibody drugs history					
No [*n* (%)]	82 (58.16)	52 (64.20)	30 (50.00)	3.274^c^	0.195
Anticoagulant drugs [*n* (%)]	33 (23.40)	15 (18.52)	18 (30.00)		
Antiplatelet drugs [*n* (%)]	26 (18.44)	14 (17.28)	12 (20.00)		
PT (s; *M*, *IQR*)	12.00 (11.35, 12.70)	12.10 (11.4, 12.90)	11.75 (11.30, 12.50)	1.266^b^	0.205
APTT (s; *M*, *IQR*)	25.20 (23.60, 27.25)	25.40 (23.75, 27.2)	24.80 (23.53, 27.33)	0.753^b^	0.452
Fibrinogen (mg/dL; *M*, *IQR*)	3.02 (2.56, 3.79)	3.15 (2.74, 3.81)	2.96 (2.45, 3.62)	1.737^b^	0.082
LDL-C (mmol/L; *M*, *IQR*)	2.34 (1.96, 2.86)	2.33 (1.96, 2.79)	2.36 (1.95, 2.91)	−0.250^b^	0.802
Pre-NIHSS score $(\bar{x}$ ± s)	19.00 ± 8.00	19.00 ± 8.00	19.00 ± 7.00	−0.166^a^	0.869
Pre-mRS Score (*M*, *IQR*)	0 (0, 0)	0 (0, 0)	0 (0, 0)	−0.744^b^	0.457
Anterior circulation [*n* (%)]	124 (87.94)	70 (86.42)	54 (90.00)	0.417^b^	0.607
Posterior circulation [*n* (%)]	17 (12.06)	11 (13.58)	6 (10.00)		
DPT (min; *M*, *IQR*)	144.00 (110.00, 186.50)	143.00 (116.50, 179.00)	144.00 (103.25, 199.00)	−0.169^b^	0.866
ORT (min; *M*, *IQR*)	494.50 (379.50, 702.50)	571.00 (362.50, 887.50)	482.50 (388.75, 550.25)	1.468^b^	0.142
TOAST					
LAA [*n* (%)]	36 (25.53)	21 (25.93)	15 (25.00)	0.178^c^	0.915
CE [*n* (%)]	63 (44.68)	35 (43.21)	28 (46.67)		
SUE [*n* (%)]	42 (29.79)	25 (30.86)	17 (28.33)		

a t.

b U.

c X^2^.

IQR, interquartile range; M, Median; PT, prothrombin time; APTT, activated partial thromboplastin time; LDL-C, low density lipoprotein cholesterol; Pre, preoperative; NIHSS, national institute of health stroke scale; mRS, modified rankin scale; ASPECTS, alberta stroke program early CT score; DPT, door-to-puncture time; Trial of Org 10,172 in Acute Stroke Treatment (TOAST); LAA, large-artery atherosclerosis; CE, cardiogenic embolism; SUE, stroke of undetermined etiology.

### Pathology of thrombosis

**Figure 1:**
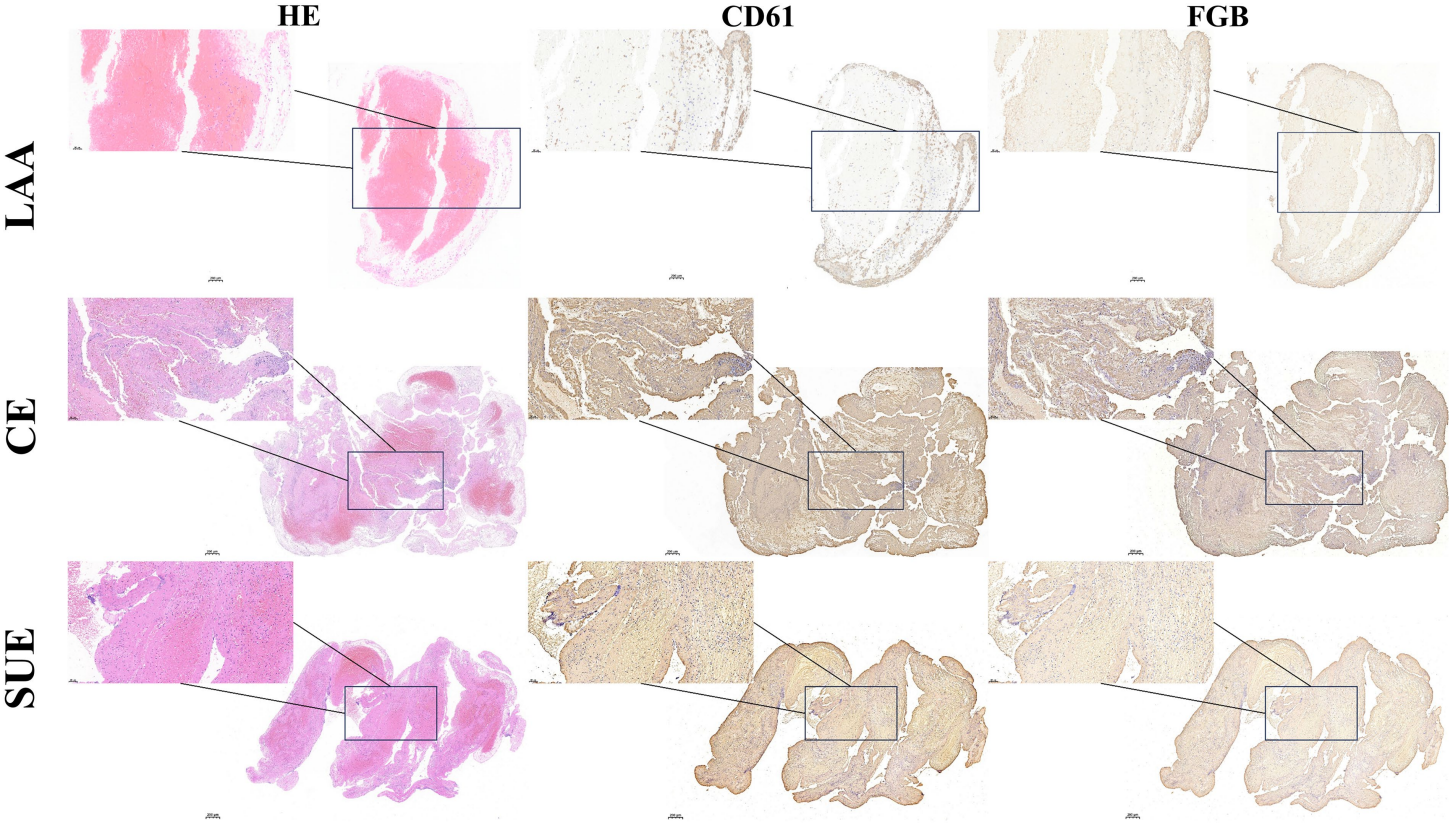
Comparison of three staining methods in different TOAST classification. We selected three representative thrombus slice samples and observed them under 100x and 400x magnification. By selecting a representative region and magnifying it 400 times. In HE staining, red represents RBC, blue represents WBC, and the rest are PLT + FIB complexes. In IHC staining, in CD61, brown indicates PLT, blue indicates WBC, and the rest are other complexes; in FGB, brown indicates FIB, blue indicates WBC, and the rest are other complexes; We found that fibrin was present in the areas rich in red blood cells. LAA, large-artery atherosclerosis; CE, cardiogenic embolism; SUE, stroke of undetermined etiology; CD61, cluster of differentiation 61; IHC, immunohistochemistry; RBC, red blood cell; PLT, platelet; FIB, fibrin; Original magnification: 100×, Scale bar = 200 μm; Total magnification: 400×, Scale bar = 50 μm.

According to HE and immunohistochemical staining, it can be seen that the thrombus is composed of RBC, a small amount of WBC, PLT and FIB. The distribution of each component in the thrombus varies depending on the stroke etiology classification. In LAA thrombus, RBC is aggregated in the center of the thrombus, with FIB and PLT distributed around the edge. It was also found that FIB is distributed in RBC aggregation area. While in CE, fibrin and platelets are evenly distributed throughout the thrombus. SUE shows similar characteristics to CE, as shown in Figure 1.

### Thrombus component analysis

**Figure 2:**
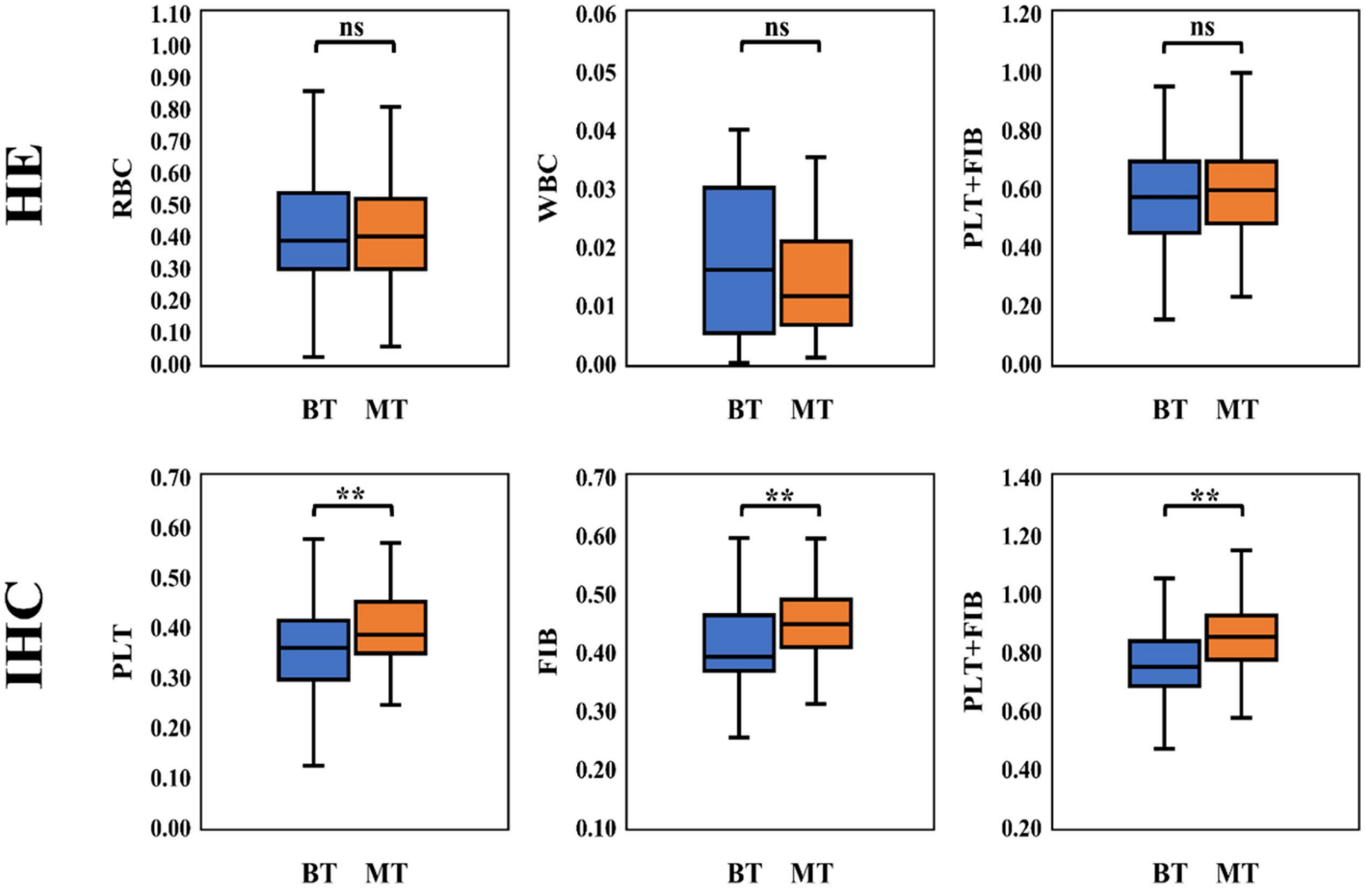
Thrombus component analysis using two distinct staining Methods in both MT group and BT group. *p < 0.05; ns: *p* > 0.05; IHC, immunohistochemistry; RBC, red blood cell; PLT, platelet; FIB, fibrin; WBC, white blood cell; BT, bridging therapy; MT, mechanical thrombectomy.

**Figure 3:**
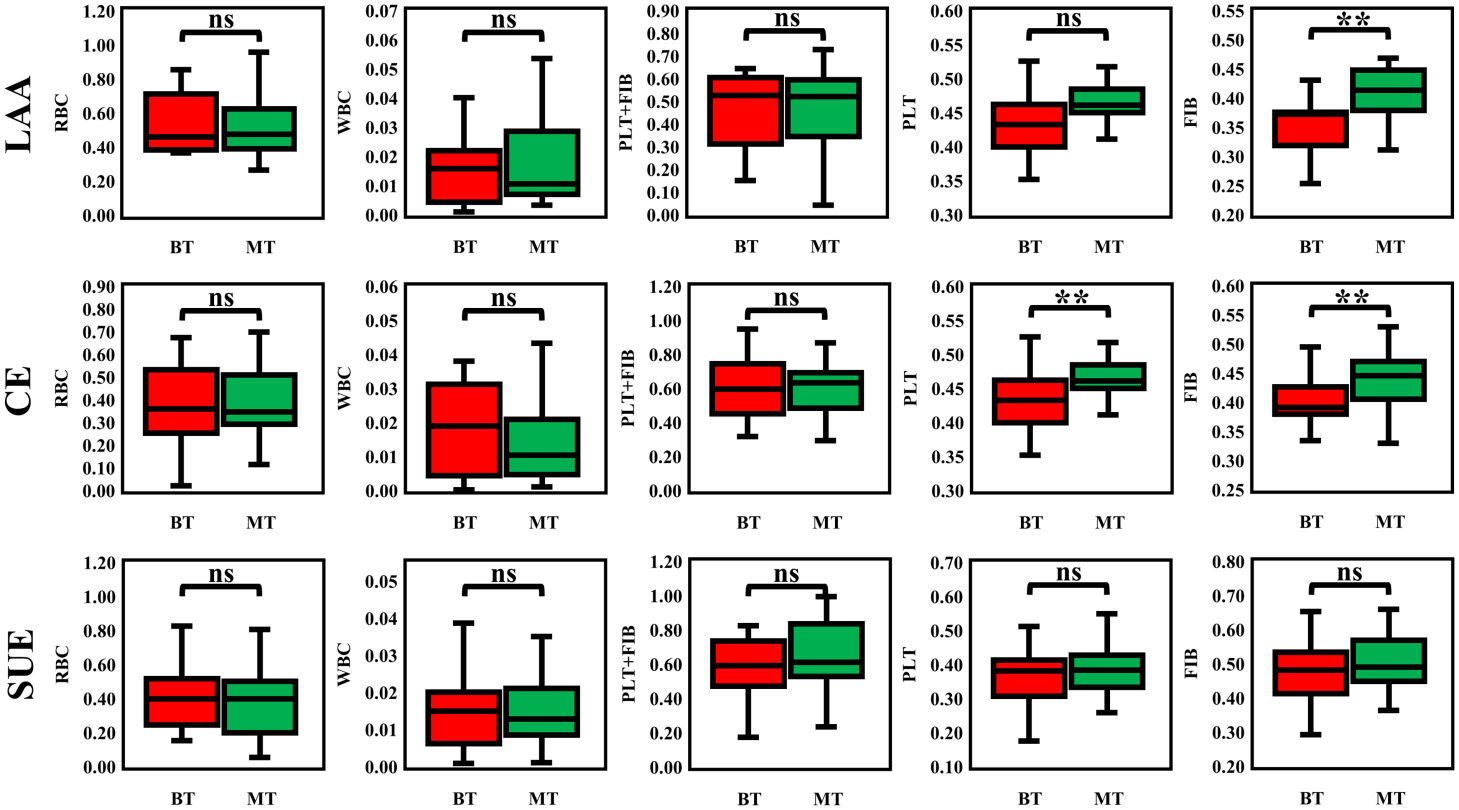
Differences in thrombus composition among AIS subtypes: LAA, CE, and SUE. **p < 0.05; ns: *p* > 0.05; LAA, Large-artery Atherosclerosis; CE, Cardiogenic Embolism; SUE, Stroke of Undetermined Etiology; RBC, red blood cell; PLT, platelet; FIB, fibrin; WBC, white blood cell; r-tPA, Recombinant Tissue Plasminogen Activator.

**Figure 4:**
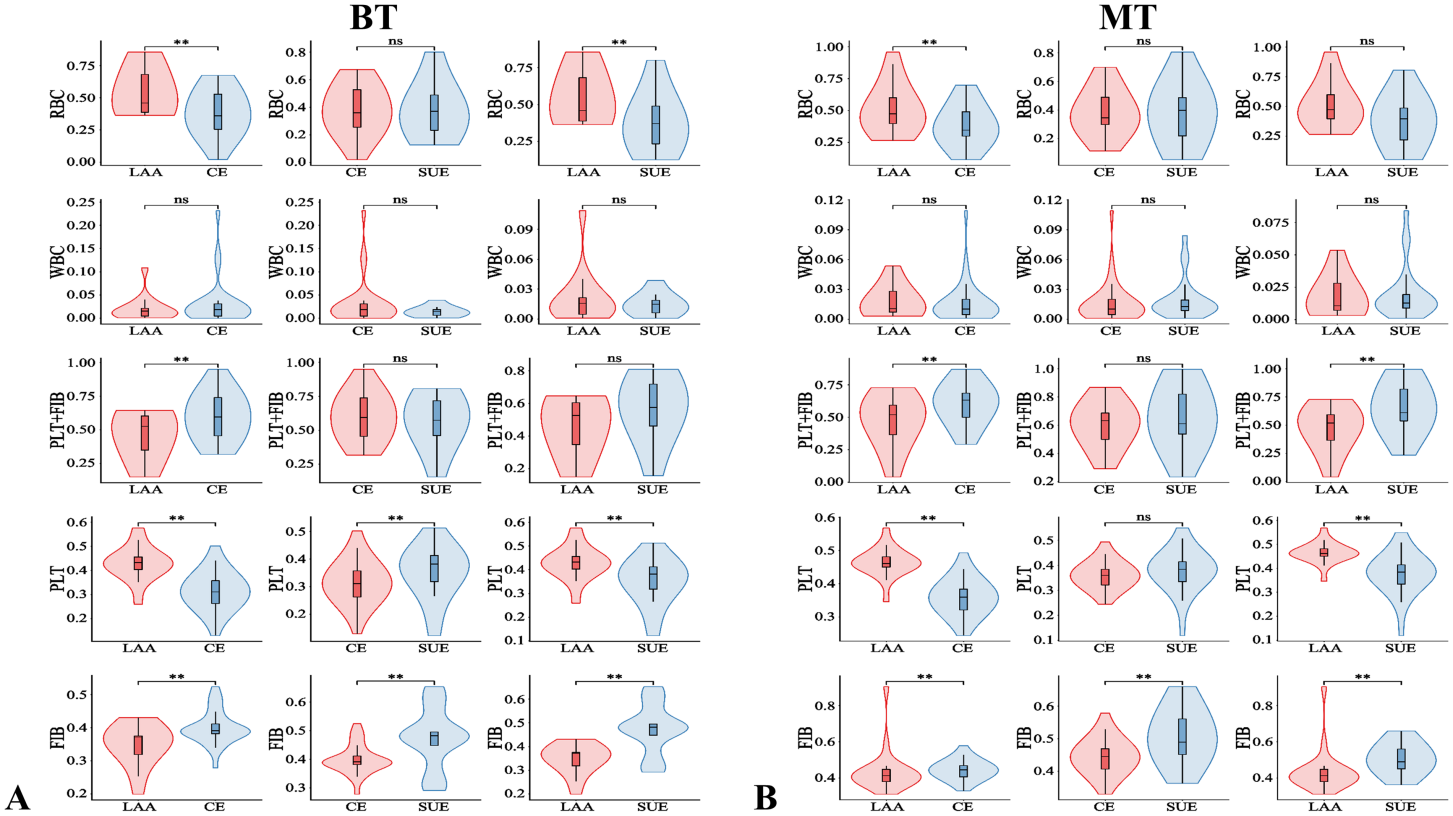
Comparison of different TOAST classifications in the BT and MT group. **p < 0.05; ns: *p* > 0.05; BT, bridging therapy; MT, mechanical thrombectomy; TOAST classification (each classification represents a group): LAA, large-artery atherosclerosis; CE, cardiogenic embolism; SUE, stroke of undetermined etiology; RBC, red blood cell; PLT, platelet; FIB, fibrin; WBC, white blood cell; PLT+FIB, PLT+FIB complex.

The baseline data of 141 patients are shown in Table 1. There were differences in the ratios of PLT and FIB between the MT group and the BT group: PLT was [38.41% (34.67, 45.19) vs. 35.82% (29.33, 41.31), *p* = 0.006], FIB was [44.73% (40.66, 49.18) vs. 39.13% (36.70, 46.78), *p* < 0.001], and the immunohistochemical PLT + FIB was [85.11% (77.01, 92.48) vs. 74.88% (67.56, 83.90), *p* < 0.001], as shown in Figure 2. The comparison results of MT and BT under different TOAST classifications are presented in Table 2 and Figure 3. The comparison of different TOAST classifications within each group of MT and BT is detailed in Table 3 and Figure 4.

**Table 2:** Comparison of mechanical thrombectomy versus bridging Therapy across different TOAST subtypes.

Group type	TOAST classification	HE			IHC	
		RBC [*n* (%); M; IQR]	WBC [*n* (%); M; IQR]	PLT+FIB [*n* (%); M; IQR]	PLT [*n* (%); M; IQR]	FIB [*n* (%); M; IQR]
LAA (*n* = 36)	MT (*n* = 15)	47.37 (38.84, 62.40)	1.06 (0.69, 2.86)	51.97 (34.36, 59.38)	46.11 (44.99, 48.44)	41.30 (37.89, 44.80)
	BT (*n* = 21)	45.96 (38.34, 71.09)	1.58 (0.43, 2.19)	52.56 (31.08, 60.41)	43.23 (39.95, 46.20)	37.22 (31.83, 37.55)
CE (*n* = 63)	MT (*n* = 35)	34.54 (29.23, 50.97)	1.03 (0.46, 2.09)	63.20 (48.41, 69.18)	36.00 (31.98, 38.41)	44.53 (40.51, 46.94)
	BT (*n* = 28)	36.00 (25.18, 53.26)	1.89 (0.43, 3.12)	59.44 (45.03, 74.42)	31.05 (26.13, 35.95)	39.13 (37.92, 42.65)
SUE (*n* = 42)	MT (*n* = 25)	39.73 (24.40, 51.56)	1.490 (0.62, 2.01)	59.00 (47.02, 73.48)	38.13 (30.65, 41.28)	48.18 (41.28, 53.36)
	BT (*n* = 17)	39.73 (19.76, 50.16)	1.280 (0.85, 2.11)	61.03 (52.81, 83.59)	38.38 (33.27, 42.70)	48.95 (44.87, 56.86)

IQR, interquartile range; M, Median; BT, bridging therapy; MT, mechanical thrombectomy; LAA, large-artery atherosclerosis; CE, cardiogenic embolism; SUE, stroke of undetermined etiology.

**Table 3:** Comparative analysis of different TOST subtypes between MT and BT groups.

Group type	TOAST classification	HE			IHC	
		RBC [*n* (%); M; IQR]	WBC [*n* (%); M; IQR]	PLT+FIB [*n* (%); M; IQR]	PLT [*n* (%); M; IQR]	FIB [*n* (%); M; IQR]
MT (*n* = 81)	LAA (*n* = 21)	47.37 (38.84, 62.40)	1.06 (0.69, 2.86)	51.97 (34.46, 59.38)	46.11 (44.99, 48.44)	41.30 (37.89, 44.80)
	CE (*n* = 35)	34.54 (29.23, 50.97)	1.03 (0.46,2.09)	63.20 (48.41, 69.18)	36.00 (31.98, 38.41)	44.53 (40.51, 46.94)
	SUE (*n* = 25)	39.73 (19.76, 50.16)	1.28 (0.85, 2.11)	61.03 (52.81, 83.59)	38.38 (33.27, 42.70)	48.95 (44.87, 56.86)
BT (*n* = 60)	LAA (*n* = 15)	45.96 (38.34, 71.09)	1.58 (0.43, 2.19)	52.56 (31.08, 60.41)	43.23 (39.95, 46.20)	37.22 (31.83, 37.55)
	CE (*n* = 28)	36.00 (25.18, 53.26)	1.89 (0.43, 3.12)	59.44 (45.03, 74.42)	31.05 (26.13, 35.95)	39.13 (37.92, 42.65)
	SUE (*n* = 17)	37.13 (21.80, 48.96)	1.49 (0.62, 2.01)	57.40 (45.42, 71.88)	38.13 (30.65, 41.28)	48.18 (41.28, 53.36)

IQR, interquartile range; M, Median; BT, bridging therapy; MT, mechanical thrombectomy; LAA, large-artery atherosclerosis; CE, cardiogenic embolism; SUE, stroke of undetermined etiology.

### Data analysis of clinical outcomes and surgical characteristics

The comparison results of MT and BT in different TOAST subtypes are detailed in Table 4. Pairwise comparisons of different TOAST subtypes within each group of MT and BT did not reveal significant differences (*p* > 0.05). The specific data are presented in Table 5.

**Table 4:** Comparative outcomes of mechanical thrombectomy vs. bridging therapy across different TOAST subtypes.

Outcome	LAA		*p*	CE		*p*	SUE		*p*
	MT (*n* = 21)	BT (*n* = 15)		MT (*n* = 35)	BT (*n* = 28)		MT (*n* = 22)	BT (*n* = 15)	
PRT (min; M; IQR)	45.00 (38.50,60.50)	55.00 (42.00,85.00)	0.101	57.00 (34.00,87.00)	69.00 (50.25,89.50)	0.125	59.00 (47.00,91.00)	60.00 (52.00,86.00)	0.949
90d mRS favorable [*n* (%)]	9 (42.86)	9 (60.00)	0.310	16 (45.71)	11 (39.29)	0.608	10 (40.00)	8 (47.06)	0.650
90d mortality [n (%)]	1 (4.76)	2 (13.33)	0.760*	6 (17.14)	4 (14.29)	1.000*	3 (12.00)	2 (11.76)	1.000*
mTICI favorable [*n* (%)]	19 (90.47)	14 (93.33)	1.000*	29 (82.86)	25 (89.29)	0.717*	22 (88.00)	15 (88.24)	1.000*
NTP (number; M; IQR)	1.00 (1.00,2.00)	1.00 (1.00,2.00)	0.682	1.00 (1.00,2.00)	1.00 (1.00,2.00)	0.717	1.00 (1.00,2.00)	2.00 (1.00,3.00)	0.317
FPY [*n* (%)]	16 (76.19)	10 (66.67)	0.801*	21 (60.00)	15 (53.57)	0.608	19 (76.00)	11 (64.71)	0.655*
SICH [*n* (%)]	1 (4.76)	2 (13.33)	0.760*	2 (5.71)	5 (17.86)	0.262	2 (8.00)	2 (11.76)	1.000*

^*^Fisher exact test; IQR, interquartile range; M, Median; BT, bridging therapy; MT, mechanical thrombectomy; LAA, large-artery atherosclerosis; CE, cardiogenic embolism; SUE, stroke of undetermined etiology; NTP, Number of Thrombectomy Procedures; 90d mRS favorable: mRS ≤ 2; mTICI favorable: mTICI grade≥2b; SICH, Symptomatic Intracranial Hemorrhage; FPY, First-pass yield.

**Table 5:** Comparative outcomes of different TOST subtypes between MT and BT groups.

Outcome	MT (81)			BT (n = 60)		
	LAA (*n* = 21)	CE (*n* = 35)	SUE (*n* = 25)	LAA (*n* = 15)	CE (*n* = 18)	SUE (*n* = 17)
PRT (min; M; IQR)	45.00 (38.50, 60.50)	57.00 (34.00, 87.00)	59.00 (47.00, 91.00)	55.00 (42.00, 85.00)	69.00 (50.25, 89.50)	60.00 (52.00, 86.00)
90d mRS favorable [*n* (%)]	9 (42.86)	16 (45.71)	10 (40.00)	9 (60.00)	11 (39.29)	8 (47.06)
90d mortality [*n* (%)]	1 (4.76)	6 (17.14)	3 (12.00)	2 (13.33)	4 (14.29)	2 (11.76)
mTICI favorable [*n* (%)]	19 (90.47)	29 (82.86)	22 (88.00)	14 (93.33)	25 (89.29)	15 (88.24)
NTP (number; M; IQR)	1.00 (1.00, 2.00)	1.00 (1.00, 2.00)	1.00 (1.00, 2.00)	1.00 (1.00, 2.00)	1.00 (1.00, 2.00)	1.00 (1.00,3.00)
FPY [*n* (%)]	16 (76.19)	21 (60.00)	19 (76.00)	10 (66.67)	15 (53.57)	11 (64.71)
SICH [*n* (%)]	1 (4.76)	2 (5.71)	2 (8.00)	2 (13.33)	5 (17.86)	2 (11.76)

IQR, interquartile range; M, Median; BT, bridging therapy; MT, mechanical thrombectomy; LAA, large-artery atherosclerosis; CE, cardiogenic embolism; SUE, stroke of undetermined etiology; NTP, Number of Thrombectomy Procedures; 90d mRS favorable: mRS ≤ 2; mTICI favorable: mTICI grade≥2b; SICH, Symptomatic Intracranial Hemorrhage; FPY, First-pass yield.

## Discussion

This study retrospectively analyzed thrombus samples from AIS patients undergoing MT, using HE staining and IHC to systematically characterize thrombus composition across TOAST subtypes (LAA, CE, SUE), assess the impact of IVT on clot composition, and evaluate clinical outcomes and surgical characteristics. The discussion focuses on key findings, underlying mechanisms, clinical implications, and research value.

Firstly, this study is the first to evaluate the impact of IVT on the composition of thrombus in different etiological subtypes by IHC labeling of PLT and FIB. The core findings are as follows: (1) IVT reduces the FIB content in LAA and CE thrombi; (2) the effect of IVT on PLT is only observed in CE thrombi; (3) there is no difference in the content of PLT and FIB in the SUE subgroup after IVT. In addition, in the LAA subtype, thrombi are characterized by a high content of RBC and a small amount of FIB and PLT. The density of the FIB network is lower than that in the CE subtype, allowing drugs to penetrate more easily (Kim Y. D. et al., 2015; Choi et al., 2018), making it more sensitive to rt-PA and leading to FIB dissolution, but no significant change was observed in the PLT component. However, this result is not entirely consistent with our expected hypothesis: we speculated that in the LAA subtype, due to the faster carotid blood flow, platelets might be washed away by the blood flow after fibrinolysis, but this phenomenon was not observed in this study, which might be related to the limited sample size. Notably, previous studies using HE staining failed to confirm a reduction in PLT + FIB in thrombus after thrombolysis. This study, through IHC technology, found that the effect of IVT on PLT may be underestimated by traditional HE staining-HE staining can only show PLT + FIB, while IHC can specifically distinguish PLT from FIB. The results show that the PLT content in CE thrombi significantly decreases after IVT. The above research results indicate that in CE-type thrombi, when exposed to r-tPA, fibrin in the marginal area (i.e., the side in contact with blood flow) dissolves first, thereby indirectly exposing and partially removing the surface platelets. This phenomenon is consistent with the findings of Doche et al. (2024). However, due to the existence of a densely cross-linked fibrin network within the thrombus (Jiang et al., 2021), its sensitivity to r-tPA is significantly reduced, showing a certain degree of resistance. This structural characteristic may be the main reason why the FIB content in CE-type thrombi in this study only decreased by 10–15%, with a limited reduction. In addition, the high FIB content and dense mesh structure of SUE thrombi may be the main reasons for their resistance to IVT, consistent with the reports of Vandelanotte et al. (2024a,b). A study (Marta-Enguita et al., 2024) has revealed that aged thrombi might develop a more compact fibrin network as a result of ongoing cross-linking reactions (such as the effect of FXIIIa). This, consequently, gives rise to resistance to thrombolysis.

Secondly, through quantitative IHC analysis, this study also found that LAA thrombi are characterized by RBC aggregation as the core feature, with RBCs mainly distributed in the central area of the thrombus, while FIB and PLT form a relatively simple structure at the periphery. Our findings are consistent with previous reports showing comparable thrombus composition patterns (Bembenek et al., 2017; Fitzgerald et al., 2021). This feature may be related to the formation mechanism of LAA thrombi - after the rupture of atherosclerotic plaques, local blood flow stasis leads to the retention and aggregation of RBC, while PLT and FIB mainly participate in the initial thrombus formation on the surface of the plaque. In contrast, CE thrombi present a “FIB-dominant” complex grid structure, with FIB and PLT widely interwoven throughout the thrombus, while RBC are scattered. This result supports the classic theory that LAA thrombi have a higher proportion of RBC and that cardiogenic emboli are mainly composed of PLT + FIB (Kim S. K. et al., 2015; Ahn et al., 2016; Maekawa et al., 2018; Fitzgerald et al., 2021), but it contradicts the earlier view that LAA thrombi are mainly composed of PLT + FIB (Packham and Mustard, 1986; White, 1994; Shin et al., 2018). Interestingly, we found that fibrin also exists in areas rich in red blood cells, which is consistent with previous studies (Staessens et al., 2020; Pir et al., 2022), suggesting that there may be interactions among these components. In RBC-dominated thrombi, a small amount of fibrin acts as a “scaffold” to wrap around red blood cells, maintaining the overall structural integrity of the thrombus. This mixed structure enhances stability (Doche et al., 2024). Previous studies only found that in HE staining, the FIB and RBC areas had relatively distinct manifestations (Staessens et al., 2020). Additionally, this study further quantified through immunohistochemistry that the PLT content in LAA thrombi was significantly higher than that in CE and SUE thrombi, which may be related to more intense local PLT activation after plaque rupture; these results are consistent with the conclusions of Fitzgerald and Brinjikji (2019) and Dargazanli et al. (2020). CE and SUE thrombi have a higher FIB content, suggesting that FIB plays a key role in the stability of the structure of CE and SUE thrombi. Moreover, we found that the component characteristics of SUE thrombi are between LAA and CE: their RBC, WBC, and PLT + FIB composition is similar to that of CE (Sporns et al., 2017; Nouh et al., 2020), but the FIB content differs between the MT and BT groups (higher in the SUE group), and only in the BT group is there a difference in PLT content between CE and SUE. This result suggests that SUE thrombi may have “mixed” pathological features, and the complexity of their etiology (such as cryptogenic embolism, small vessel disease, or multi-site embolism) may lead to the heterogeneity of thrombus components. However, the current understanding of the specific mechanisms (such as the thrombus formation microenvironment and dynamic evolution process) is still limited, and further subgroup analysis and mechanism exploration are needed. In patients with TOAST classification, the comparison between MT and BT showed that the PRT in the MT group tended to decrease, while FPY showed the opposite trend. We analyzed that the possible mechanism might be that intravenous thrombolysis led to partial fibrinolysis, damaged the structure of thrombus, produced thrombus fragments, and then thrombus escape might occur, making it impossible to completely remove the thrombus at one time, thus prolonging the operation time. Doche et al.’s (2024) study also pointed out a similar conclusion. In addition, the application of thrombolytic drugs may change the structural stability of thrombus, which is also an important factor affecting complete thrombus removal. Although Jadhav et al.’s (2022) study found that FPY was important for a good prognosis, no difference was found in our study, which might be related to the sample size. No difference was found in other prognostic indicators, suggesting that for patients with different TOAST classifications, the two treatment strategies of MT and BT did not show significant differences in overall efficacy. Previous studies (Doheim et al., 2025) did not involve the differences in clinical outcomes of TOAST subtype patients after receiving MT or BT. This study to some extent filled the gap in this field, but large-scale clinical trials are still needed to further verify the above conclusions.

Finally, the innovation of this study lies in the combined application of HE staining and IHC techniques, which overcomes the limitations of single staining methods: although HE staining can display PLT + FIB, it cannot precisely distinguish PLT from FIB (Sporns et al., 2017; Maekawa et al., 2018; Gong et al., 2019; Patel et al., 2021; Manisha et al., 2024); Masson staining has limited ability to differentiate the two; while IHC achieves precise quantification of components through specific labeling, providing a new tool for analyzing the microstructure of thrombi (Brinjikji et al., 2021). Meanwhile, systematically correlating the changes in thrombus composition with the effect of vascular recanalization, surgical conditions, and patient functional outcomes further enhanced the clinical depth of the research. It is worth noting that in the acquisition of thrombi, we selected the first thrombus from each case for study, and almost all of them were obtained through the first or second thrombectomy, reducing the potential changes in thrombus composition due to multiple thrombectomies. Many previous studies (Boeckh-Behrens et al., 2016; Vandelanotte et al., 2024a) did not describe the method of thrombus selection, such as whether the thrombi were randomly selected from different thrombectomy procedures or whether all thrombi from each patient were comprehensively quantified. In such cases, the results obtained might be biased. Of course, intravenous thrombolysis itself may affect the structure and composition of thrombi, and this confounding factor is difficult to completely avoid in clinical research. However, as a multi-center retrospective analysis, this study has the following limitations: (1) The sample size is small, which may reduce the statistical power of subgroup analysis (e.g., the IVT effect on SUE thrombi did not reach significance); (2) The retrospective design cannot completely eliminate selection bias (e.g., some patients did not receive IVT due to contraindications); (3) Thrombus samples were only from MT patients and did not include patients who received only IVT or did not receive thrombolysis, thus failing to fully reflect the dynamic evolution of thrombus components; (4) Potential thrombogenic components such as vWF and extracellular DNA were not stained, making it impossible to verify their association with IVT resistance [e.g., the hypothesis of elevated PAI-1 levels proposed by Jiang et al., 2021]. Future large-scale, multicenter prospective studies should integrate thrombus component parameters with multidimensional imaging features (e.g., thrombus volume, embolization site, structural stability) and key clinical indicators to build a multimodal prognostic prediction model. This model can guide clinical decisions—such as selecting thrombolytic therapy or prioritizing mechanical thrombectomy based on specific thrombus composition—enabling precise patient stratification and individualized treatment optimization. Additionally, further research is needed on the dynamic evolution of thrombus components and novel thrombolytic targets (e.g., enzymes that degrade dense fibrin networks) to improve recanalization rates and clinical outcomes in stroke patients.

In conclusion, this study precisely quantified the component characteristics of thrombi in different etiological subtypes and their associations with IVT and clinical outcomes, revealing the selective impact of IVT on thrombus composition. This provides important theoretical support for optimizing the combined strategy of intravenous thrombolysis and mechanical thrombectomy in AIS patients.

## Conclusion

Overall, thrombus composition differs between LAA and CE regardless of IVT: LAA is red blood cell–rich, whereas CE is fibrin-dominant; SUE resembles CE but has higher FIB content. IVT benefits AIS-LVO patients by reducing FIB, particularly in LAA, while PLT levels remain higher in LAA than in CE or SUE. FIB is present in both PLT-rich and RBC-rich regions, suggesting multiple roles in thrombosis. By loosening the clot structure, IVT may improve thrombectomy success but could cause fragment embolization or prolong procedure time. Although no differences in MT vs. BT outcomes were observed across TOAST subtypes in this study, larger studies are warranted.

## Data Availability

The datasets supporting the conclusions of this article will be made available by the authors, without undue reservation.
